# Interdisciplinary Synergy to Reveal Mechanisms of Annexin-Mediated Plasma Membrane Shaping and Repair

**DOI:** 10.3390/cells9041029

**Published:** 2020-04-21

**Authors:** Poul Martin Bendix, Adam Cohen Simonsen, Christoffer D. Florentsen, Swantje Christin Häger, Anna Mularski, Ali Asghar Hakami Zanjani, Guillermo Moreno-Pescador, Martin Berg Klenow, Stine Lauritzen Sønder, Helena M. Danielsen, Mohammad Reza Arastoo, Anne Sofie Heitmann, Mayank Prakash Pandey, Frederik Wendelboe Lund, Catarina Dias, Himanshu Khandelia, Jesper Nylandsted

**Affiliations:** 1 Niels Bohr Institute, University of Copenhagen, DK-2100 Copenhagen, Denmark; florentsen@nbi.ku.dk (C.D.F.); moreno@nbi.dk (G.M.-P.); helena.danielsen@nbi.ku.dk (H.M.D.); mohammadreza.arastoo@nbi.ku.dk (M.R.A.); 2 Department of Physics, Chemistry and Pharmacy, University of Southern Denmark, DK-5230 Odense, Denmark; mularski@sdu.dk (A.M.); zanjani@sdu.dk (A.A.H.Z.); klenow@sdu.dk (M.B.K.); pandey@sdu.dk (M.P.P.); fwl@sdu.dk (F.W.L.); 3 Membrane Integrity, Cell Death and Metabolism Unit, Center for Autophagy, Recycling and Disease, Danish Cancer Society Research Center, DK-2100 Copenhagen, Denmark; swantjechristinhaeger@gmail.com (S.C.H.); stilau@cancer.dk (S.L.S.); anhe@cancer.dk (A.S.H.); cad@cancer.dk (C.D.); 4 Department of Cellular and Molecular Medicine, Faculty of Health Sciences, University of Copenhagen, DK-2200 Copenhagen, Denmark

**Keywords:** annexin, plasma membrane repair, membrane curvature, membrane curvature sensing, membrane shaping, interdisciplinary research, cell rupture, membrane damage

## Abstract

The plasma membrane surrounds every single cell and essentially shapes cell life by separating the interior from the external environment. Thus, maintenance of cell membrane integrity is essential to prevent death caused by disruption of the plasma membrane. To counteract plasma membrane injuries, eukaryotic cells have developed efficient repair tools that depend on Ca^2+^- and phospholipid-binding annexin proteins. Upon membrane damage, annexin family members are activated by a Ca^2+^ influx, enabling them to quickly bind at the damaged membrane and facilitate wound healing. Our recent studies, based on interdisciplinary research synergy across molecular cell biology, experimental membrane physics, and computational simulations show that annexins have additional biophysical functions in the repair response besides enabling membrane fusion. Annexins possess different membrane-shaping properties, allowing for a tailored response that involves rapid bending, constriction, and fusion of membrane edges for resealing. Moreover, some annexins have high affinity for highly curved membranes that appear at free edges near rupture sites, a property that might accelerate their recruitment for rapid repair. Here, we discuss the mechanisms of annexin-mediated membrane shaping and curvature sensing in the light of our interdisciplinary approach to study plasma membrane repair.

## 1. Introduction

Membranes of eukaryotic cells play fundamental roles in organizing cellular compartments and cluster specific molecules to generate signaling platforms. Beyond defining the physical cell boundary, the plasma membrane serves a dual purpose: it allows the communication and exchange of solutes with the extracellular environment, while guarding the cell from its surroundings. In addition, the plasma membrane facilitates cell–cell interactions and adhesion and regulates the architecture of the cell. Thus, it is crucial that the plasma membrane is kept intact to ensure cell survival.

The plasma membrane is composed of both structural and signaling lipids, as well as embedded proteins. The major structural lipids are glycerophospholipids (such as phosphatidylserine and phosphatidylcholine), sterols, and sphingolipids (e.g., sphingomyelin) [1]. Of note, the enrichment of sphingolipids and sterols, like cholesterol, aids in resisting mechanical stress [2]. Plasma membrane topology is shaped by the geometrical properties of its lipids, in combination with both integrated and peripheral proteins, including components of the cytoskeleton [3]. Glycerophospholipids with small head groups, such as phosphatidylethanolamines and phosphatidic acid, are cone-shaped and have the propensity to induce membrane curvature, thus lipid shape is intimately related to membrane shape [3,4]. Moreover, the surface charge of membranes influences its interaction with proteins, including curvature inducing regulators, and is determined by the head group charge of individual lipids [5,6]. This is exemplified by the family of annexin proteins that are characterized by their capacity to bind negatively charged phospholipids in a Ca^2+^-dependent manner, to exert their various functions in membrane organization, trafficking, and repair [7].

Unlike prokaryotic cells, which are shielded by a cell wall, mammalian cells are primarily protected by their plasma membrane and appear more susceptible to cell injuries. To counteract the eminent threat that membrane damage would impose on cellular homeostasis, robust repair mechanisms have evolved to ensure that membrane holes are sealed within the timeframe of tens-of-seconds to maintain cell integrity [8,9]. If disruptions to the plasma membrane are not repaired rapidly, the resultant pronounced osmotic and ionic imbalances would lead to cell death. Skeletal muscle and cardiac muscle cells are classical examples of cells that, due to their high mechanic activity, need to cope with extensive plasma membrane disruptions [10]. Similarly, epithelial cells in the gut and skin fibroblasts are exposed to both mechanical and chemical stimuli and experience regular wounding [11]. However, it seems that all cell types can potentially experience plasma membrane injury and have the capacity to repair lesions [12].

Deficiency in plasma membrane repair is associated with several diseases. The best established link between repair deficiency and disease phenotype is observed in muscular dystrophies, such as dysferlin-deficient muscular dystrophy, where a failure to repair repeated membrane lesions leads to progressive muscle wasting [13]. Inversely, our research supports that enhanced plasma membrane repair plays a role in helping metastatic breast cancer cells cope with the physical stress imposed during invasion [14,15].

The importance of Ca^2+^ for membrane repair was recognized in 1930 by Heilbrunn, who discovered that sea urchin eggs failed to repair upon mechanical damage when the surrounding solution was depleted of Ca^2+^ [16]. Despite the scarce knowledge regarding membranes and cell compartmentalization at the time, this discovery pointed to Ca^2+^ as a fundamental trigger of plasma membrane repair. In intact resting cells the intracellular free Ca^2+^ concentrations in the cytoplasm are in the nanomolar range (~100 nM), while extracellular Ca^2+^ concentrations are in the millimolar range [17]. This steep Ca^2+^ gradient is important for signaling events and is established by actively pumping Ca^2+^ into the extracellular space and intracellular Ca^2+^ stores, which is also crucial to prevent Ca^2+^ cytotoxicity. Upon plasma membrane injury, the Ca^2+^ gradient leads to a rapid and pronounced flux of Ca^2+^ into the cell’s cytoplasm, which poses a threat to the cell [18,19]. However, this rise in Ca^2+^ acts at the same time as the triggering event for plasma membrane repair mechanisms.

## 2. Annexins in Repair

Several functions have been reported for annexins, and most of them include their involvement in membrane trafficking events [20]. Interestingly, during the last two decades more evidence regarding their role in plasma membrane repair has emerged [20,21,22,23]. Most proteins that play a role in plasma membrane repair have the ability to bind Ca^2+^ and become activated upon injury-induced Ca^2+^ influx. However, the recruitment of repair components to the injured plasma membrane can also be facilitated via complex formation with other Ca^2+^-binding proteins. This is exemplified by recent data showing a link between the Ca^2+^-dependent, phospholipid-binding protein annexin A7 (ANXA7), and the shedding of damaged membrane during repair facilitated by the endosomal sorting complex required for transport III (ESCRT-III) complex [22]. In detail, ANXA7 localizes to the injury site where it forms a complex with apoptosis-linked gene 2 (ALG-2) and ALG-2-interacting protein X (ALIX) [23]. ANXA7 facilitates thereby anchoring of ALG-2 to the damaged plasma membrane, where ALG-2 can assemble the ESCRT-III complex and drive shedding of damaged membrane [22].

Accumulating evidence indicates that annexins are instrumental for coping with plasma membrane injuries, although their exact mode of action is not well characterized. Annexins are a family of proteins (in mammals: ANXA1-11 and ANXA13) which share a structurally conserved C-terminal domain and the functional property of being able to bind anionic phospholipids in a Ca^2+^-dependent manner. Binding to membranes is mediated by their core domain, which consists of four characteristic structural annexin repeats [24]. The N-terminal domain varies in length and composition and is responsible for the interaction with other proteins, giving rise to different functional properties [24].

Work on dysferlin-deficient mouse muscle cells pioneered the hypothesis that annexins function in plasma membrane repair. ANXA1 and ANXA2 were found to associate with dysferlin upon muscle cell membrane injury. Since ANXA1 and ANXA2 were known to cause aggregation and fusion of liposomes in vitro [25], Lennon et al. proposed that the annexins could aid repair by facilitating fusion of vesicles with the injured plasma membrane [26]. Wounding experiments, using laser injury, confirmed that ANXA1 localizes to the injury site and that inhibition of ANXA1 function impedes plasma membrane repair [27].

Involvement of ANXA1 and ANXA6 could also be detected in microvesicle shedding of streptolysin O (SLO) pores [28]. Furthermore, data from the Protein Data Bank (PDB), where annexins have been crystallized, show that annexins have varying Ca^2+^ binding affinity, which adds another level of complexity to plasma membrane repair mechanisms. In line with this, ANXA6 was shown to have higher Ca2+ sensitivity than ANXA1, which led to differential recruitment patterns depending on the extent of the membrane lesions [28]. The importance of ANXA6 in muscle cell plasma membrane repair was recognized when mutations of the ANXA6 gene were found to negatively impact the phenotype of dysferlinopathy (a group of rare muscular dystrophies with recessive mutations in the dysferlin gene). Focal laser injury experiments revealed that ANXA6 localizes to the site of injury within seconds forming a repair cap [29,30]. Later work showed that ANXA6 localization to the injury site in muscle cells is actin-dependent and coincides with recruitment of ANXA1, ANXA2, and ANXA5 [31].

Even though the function of annexins in plasma membrane repair was initially merely explained by the ability of annexins to aid vesicle fusion, research now indicates that other mechanisms play a role for annexin-mediated wound closure. ANXA5, for example, was shown to assemble into 2D protein arrays around the injury site, preventing wound expansion [32,33]. To that end, using supported membrane models we have identified membrane binding and shaping features of other annexins that are independent of vesicle fusion events, pointing to a direct effect of annexins on membranes [34]. For example, ANXA4 was shown to generate a curvature force at free membrane edges because of its Ca^2+^-dependent homo-trimerization, while binding of ANXA6 led to a constriction force [35]. Therefore, it is likely that annexins possess different membrane-shaping properties, allowing for a tailored response that meets the needs of repair. In concert with other described processes, such as actin remodeling, annexins lead to efficient and rapid repair.

Interestingly, certain annexins, which are central components in plasma membrane repair, also display strong affinity for high membrane curvatures as we have shown recently in Moreno-Pescador et al. [36]. This suggests that membrane shaping and curvature sensing are coupled in a feedback loop where initial curvature generation by annexins leads to further recruitment of more annexins. It is therefore essential that the effect of biophysical cues, like membrane curvature, are thoroughly investigated for a comprehensive understanding of the activity and function of annexins.

## 3. Interdisciplinary Approaches to Address Annexin Function

Given their complexity and rapid dynamics, detailed mechanisms of annexin-mediated membrane shaping and repair can only be resolved by incorporating sophisticated physical methods for assessing biophysical parameters like curvature and tension. Thus, interdisciplinary research within biophysics, molecular simulations, and molecular and cellular biology is needed to understand these mechanisms. This approach proved successful, as exemplified in recent studies showing that annexin family members share a common ability of membrane curvature induction, which seems to be important for their function in repair [24,25]. Here, the impact of annexins on planar membranes with stable, free edges are relevant for studying the membrane conformation near a hole.

The annexin core domain has a convex shape at its membrane-binding interface [21]. Thus, membrane-association of annexins can generally be expected to induce spontaneous membrane curvature and possibly produce membrane shape changes. The shape changes generated by annexins depend crucially on the initial membrane geometry and on the presence of free membrane edges in particular. Thus, the use of membrane model systems prepared with specific geometries allow the systematic study of the interplay between annexin binding, membrane shape, and shape remodeling.

Supported planar membranes in a stacked conformation are prepared using spincoating with the secondary membranes existing as isolated patches on the primary membrane. The patches have stable, free edges at their borders and have minimal interactions with the solid support due to the presence of the primary membrane [26]. These membrane patches serve as useful models for the plasma membrane near the injury site and address the experimental challenge that a membrane containing a hole is often unstable and the hole is short-lived. The planar patches allow out-of-plane bending, away from the supported surface and can be used for monitoring shape changes induced by different proteins. To this end, the impact of different annexin family members on the morphology of membrane patches with free edges were recently examined [24].

Notably, the annexin members ANXA4 and ANXA5 were both found to induce rolling of the patch starting from the free membrane edges at the patch border. Complete roll-up of a cell-sized membrane patch (50–100 µm) occurs over a time scale of 5–10 s. This result shows that membrane binding of ANXA4 and ANXA5 under physiological conditions induces spontaneous curvature. The curvature generation can be expected to also occur near a plasma membrane hole when Ca^2+^ briefly enters the cytoplasm and cytosolic annexin binds to the negatively charged internal leaflet.

In order to model curvature generation around a plasma membrane hole, it has proven useful to describe the system using theoretical modeling of the membrane curvature energy.

According to the classical theory by Helfrich [27], the curvature elastic energy $H_{\mathrm{curve}}$ of a membrane with area A can be written as:

(1) $$H_{\mathrm{curve}}=\int_{A}\left[\frac{1}{2}k_{c}{\left(\bar{c}-c_{0}\right)}^{2}+k_{G}\bar{c_{G}}\right]dA$$

where $k_{c}$ is the mean curvature elastic modulus [J], $k_{G}$ is the gaussian curvature elastic modulus [J], and $c_{0}$ is the spontaneous curvature [$m^{-1}$]. The local curvature of the membrane is described by the two principal radii of curvature, $R_{1}$ and $R_{2}$. The mean curvature is defined as: $\bar{c}=\frac{1}{R_{1}}+\frac{1}{R_{2}}$ and the gaussian curvature as: $\bar{c_{G}}=\frac{1}{R_{1}}\frac{1}{R_{2}}$.

**Figure 1:**
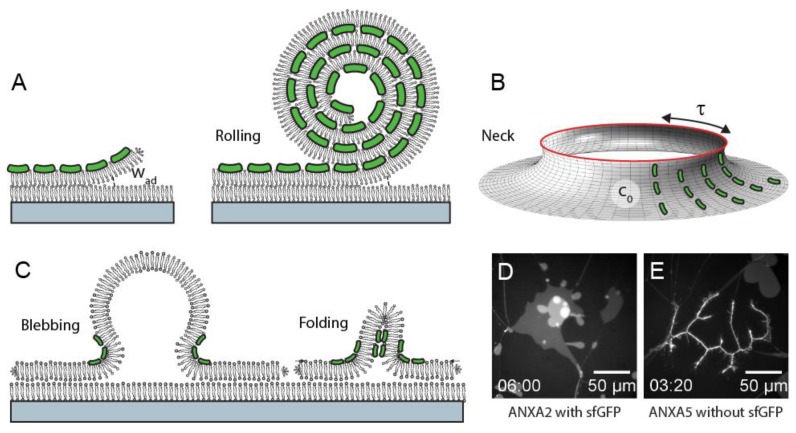
Binding of annexins (green) to a planar membrane patch with free edges and adhesion energy w_ad_ inducing spontaneous curvature and a rolling morphology of the patch (**A**). Translation to the geometry of a membrane hole (**B**) where the edge tension τ and the spontaneous curvature c_0_ acts to create a stable neck conformation. Example of blebbing/folding morphologies induced by ANXA1 and ANXA2 (**C**) and examples of fluorescence data for patches (POPC: 1-palmitoyl-2-oleoyl-sn-glycero-3-phosphocholine, POPS: (1-palmitoyl-2-oleoyl-sn-glycero-3-phospho-L-serine), 9:1 ratio, DiDC18) showing blebbing (**D**) and rolling (**E**).

*H_curve_* describes the curvature energy associated with a membrane of a general shape, for example a curved membrane near a plasma membrane hole. The spontaneous curvature c_o,_ is a quantity describing the tendency of a membrane to spontaneously curve with a curvature radius (*1/c_o_*). The effect of annexin binding to a membrane is potentially complex, but it can approximately be modeled as a non-zero value of *c_o_*. When describing a membrane containing a hole, there will additionally exist a tension force (edge tension) associated with the formation of the free edge. In a simple picture, the edge tension acts to contract the hole while the curvature energy (*H_curve_* equation) acts oppositely, by bending the membrane out-of-plane and increasing the edge radius. As previously shown [28], an equilibrium configuration is possible that balances the curvature and tension energies to create a stable neck-like shape of the membrane near the hole. We propose that ending of the membrane near a plasma membrane hole, via a mechanism as described above, plays a functional role in the plasma membrane repair process. In a cellular system, membrane re-shaping is envisioned to involve the concerted action of several annexins plus other repair proteins to rapidly bend, constrict, and finally seal the hole (Figure 1A–C).

Members of the family of human annexins were shown to induce distinctly different morphologies in the planar membrane patches [24]. This was observed despite the fact that the annexins all contain a membrane-binding core domain, which is highly conserved. In addition to large scale (cooperative) rolling as induced by ANXA4 and ANXA5, rolling in a fragmented morphology was observed for ANXA3 and ANXA13. Rolling was not observed for ANXA1 and ANXA2, which instead both induced a blebbing/folding type morphology of the membrane patch (Figure 1D,E). ANXA7 and ANXA11 induce rolling, in addition to the generation of lens-shaped membrane inclusions containing the protein and phosphatidylserine lipids. In total, the morphologies induced by annexins in membrane patches correlate well with a dendrogram of their amino acid sequences [24]. This points to an important functional role of the N-terminal annexin domain in reshaping membranes.

A deeper insight into the interplay between molecular curvatures and the rich polymorphic membrane shapes, which can be induced by annexins, will require theoretical simulations and also development of assays for studying membrane shaping in 3D, e.g., surrounding a membrane hole, and to study curvature sensing by this large class of proteins. 

More specifically, the recent developments in super-resolution microscopy, like stochastic optical reconstruction microscopy (STORM) [29] and stimulated emission depletion (STED) [30], will be valuable for investigating the shape evolution of the injured site. STORM has been used to image the cortical actin of cells with great detail [31] and could be used for resolving the rearrangement of cortical actin, which is known to regulate both plasma membrane shape and tension. Finally, faster imaging modes like STED or high speed-atomic force microscopy (AFM) could capture the steps in the formation of a hole and the subsequent membrane healing.

## 4. Annexins Are Recruited by Membrane Curvature

Proteins with the ability to shape membranes into highly curved structures often have the ability to sense high membrane curvatures. Such proteins include the well-studied Bin/amphiphysin/Rvs BAR domain containing proteins, which have all been verified as both curvature generators and sensors of specific membrane curvatures that correlate with their molecular shape [32,33,34]. The curvature sensing ability of annexins has only recently been recognized [35,36] despite the convex shape of the conserved membrane binding core domain, present in all annexins. Naively, one could expect that the convex shape, of the membrane binding domain, would imply that all annexins could be potential sensors of negative membrane curvatures. However, looking at the non-trivial curvature generation by different annexins this argument could be too simple, in particular considering the complex protein–protein interactions, variable Ca^2+^ sensitivity, and the variability in the N-terminal domains amongst the annexins.

**Figure 2:**
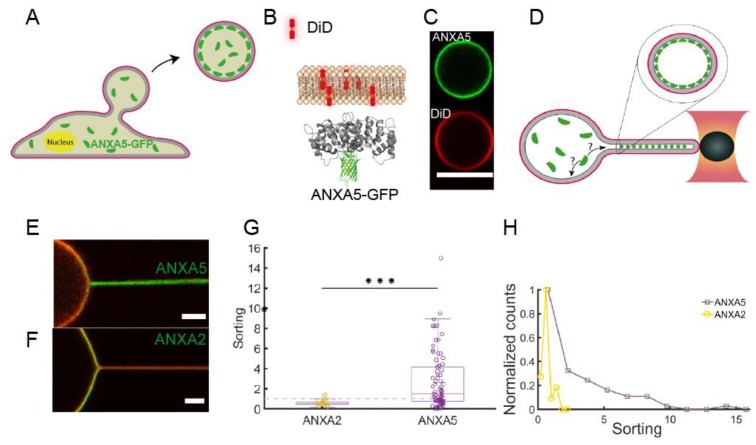
Membrane curvature sensing of ANXA2 and ANXA5 measured in lipid nanotubes extracted from Giant Plasma Membrane Vesicles (GPMVs). (**A**) GPMVs are derived from HEK293T cells expressing ANXA2-GFP or ANXA5-GFP. (**B**) GFP tagged annexins bind to the inner side of the lipophilic carbocyanine DiD labeled membrane. (**C**) Images of DiD (red) labeled GPMVs containing ANXA5-GFP (green). (**D**) Schematic of optical manipulation of the vesicles to form nanotubes with radius ~50nm and length 10µm. (**E**) Overlay image of a GPMV and nanotube containing GFP tagged ANXA5 and DiD membrane label. (**F**) Overlay image of a GPMV and nanotube containing GFP tagged ANXA2 and membrane label DiD. (**G**) Quantification of curvature sorting for ANXA2 and ANXA5, respectively. The dashed line represents Sorting = 1 corresponding to no sorting. *** *p* = 0.004. (**H**) The Sorting values from (**G**) plotted as a histogram which reveals significant heterogeneity in the Sorting by ANXA5. Reproduced with permission from [35].

Our studies, using isolated giant plasma membranes vesicles (GPMVs), have shown that both ANXA4 [36] and ANXA5 [35] are efficient sensors of negative membrane curvatures. Optical manipulation of the GPMVs was used to extract nanotubes which exhibit extreme membrane curvatures as shown in several studies on giant unilamellar vesicles (GUVs) [34]. Using GPMVs isolated from living cells expressing ANXAs coupled to GFP circumvents the step of protein encapsulation used in studies with artificial vesicles and also allows the curvature sensing to be investigated in a more physiologically-relevant environment with complex lipid and protein composition (See Figure 2A–C). Using optical manipulation, nanotubes were extracted from the vesicles (Figure 2D–F) and protein sorting between the nanotube and the vesicle membrane was quantified by the relative sorting parameter, S:

(2) $$S=\frac{{\left(I_{prot}/I_{mem}\right)}_{tube}}{{\left(I_{prot}/I_{mem}\right)}_{vesicle}}$$

where *S* is a measure of the relative density of protein on the tube (*I_prot_*)_tube_ versus the quasi-flat surface on the vesicle, (*I_prot_*)_vesicle_. The intensity from a lipid analog in the membrane, (*I_mem_*)_tube_ scales with the tube diameter and (*I_mem_*)_vesicle_ is used for normalization to account for possible different concentrations of membrane dye in different vesicles.

As shown in Figure 2E,G the density of ANXA5 was found to increase significantly within nanotubes extracted from GPMVs which were derived from HEK293T cells. The increase in density of ANXA5 ranged up to 10–15 times higher than the density on the quasi-flat region on the vesicle membrane (Figure 2G) although with significant heterogeneity in the sorting as shown in Figure 2E,G,H. Interestingly, the curvature sensing of the cognate protein ANXA2 did show a majority of sorting values below 1 indicating a slightly negative affinity for the curvatures within the nanotubes (Figure 2F–G). These results indicate that proteins from the annexin family can differ remarkably in curvature sensing despite the similarly shaped membrane binding core domain. Furthermore, we recently found that ANXA4 senses membrane curvature with a similar effect as ANXA5 and the curvature sensing was dependent on the ability of ANXA4 to form trimers [36]. The curvature sensing and membrane rolling induced by ANXA4 and ANXA5, but not ANXA2, could together indicate that trimerization is critical for membrane curvature sensing and curvature induction. Beyond these results, future experiments should test whether the two dimensional curvature of annexins can discriminate between spherical and cylindrical curvatures in similar assays as used in Li et al. [37].

Consistent with the rolling and sensing experiments discussed above multiscale simulations have revealed that ANXA4 trimers both induce and sense membrane curvatures [36]. All-atom (AA) molecular dynamics (MD) simulations provide molecular details of the interactions of annexins with lipid membranes of various compositions. Coarse-grained MD simulations, where multiple heavy atoms in a molecule are represented by single beads, are used to analyze the interactions of multiple annexin molecules with a bilayer surface, and the micron-scale scaffolding of annexins on a membrane surface [38]. Large-scale membrane conformations induced by annexins are probed by Monte-Carlo simulations, where the membrane is modeled as a fluid triangulated surface, and the annexin molecules are modeled as a vector field that induced local curvature changes in the membrane [36]. The three different computational techniques constitute an inter-coupled multi-scale simulation strategy where annexin-membrane interactions are investigated from the molecular level all the way to mesoscale fluctuations in membrane shape.

We used all-atom simulations to calculate the annexin-induced curvature upon POPC (1-Palmitoyl-2-oleoylphosphatidylcholine):POPS (1-Palmitoyl-2-oleoylphosphatidylserine) (4:1 ratio) lipid membranes in the presence of Ca^2+^. Simulations of ANXA4 reveal that the ANXA4 trimer induces a significant negative average mean curvature of 0.0024 ± 0.0002 nm^−1^ on the bilayer. The curvature is calculated by fitting the coordinates of the lipid head group phosphorus atoms by a two-dimensional Fourier series. The simulations also reveal a significant accumulation of PS lipids below the annexin trimer surface, which is to be expected because Ca^+2^ ions cross-link between anionic amino acids on the annexin trimer and anionic POPS lipids [39]. It is worth noting that although such lipid reorganization can be observed in all-atom simulations on time-scales of hundreds of nanoseconds, the diffusion of lipids in the membrane plane is slow, and is best examined by coarse-grained simulations on longer time scales. The trimer of ANXA5 has a similar impact on bilayer properties, while the effect of monomers of ANXA4 and ANXA5 differ in their interaction with the bilayer surface, compared to their trimer forms (unpublished data). Future studies along the lines described in the previous section are needed to test the theoretical predictions in experimental model systems using GUVs with controlled membrane curvatures and known protein densities.

**Figure 3:**
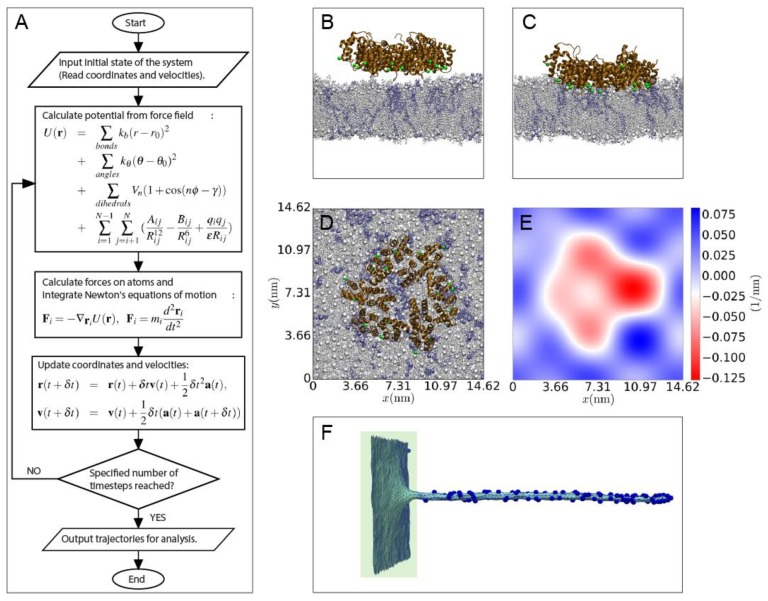
(**A**) An overview of the molecular dynamics (MD) procedure, with a simple form of force field and velocity-verlet integration algorithm. The integration timestep in all-atom MD is usually 2 fs. (**B**) Initial simulation setup of the ANXA4 trimer near a POPC:POPS (4:1) bilayer. (**C**) Final snapshot showing the indentation of the membrane. (**D**) Top view of the final snapshot. (**E**) The 2D curvature profile for a surface passing through the center of the membrane in panel C. (**F**) Monte-Carlo simulation snapshot of ANXA4 protein affinity for a membrane patch (flat) and a highly curved nanotube generated by pulling a vertex from a flat membrane. Note that the proteins are depicted on the outer surface for clarity.

The curvature calculated from the all-atom MD simulations is fed as a parameter into the Monte-Carlo simulations, which show that ANXA4 trimers accumulate on the inner membrane of highly curved nanotubes, and are desorbed from the less curved membrane surface (Figure 3). The data are in excellent agreement with the curvature-sensing experiments presented in Figure 2, which show a significant increase in the concentration of ANXA5 within nanotubes compared to the flat GPMV surface. The Monte-Carlo method described here simulates the distribution of annexins on a closed membrane surface. We are currently developing a framework of the Monte-Carlo simulations where the effect of annexins onto a free membrane edge can be investigated. Such a setup will open the possibility of investigating the effect of multiple annexins on the shape evolution of a free membrane edge, bringing us even closer to simulating the mechanism of membrane repair.

## 5. Annexin Scaffolding

Some members of the annexin family have been shown to form large scale protein scaffolds at high protein densities. These scaffolds have been imaged by atomic force microscopy (AFM) and consist of a regular lattice of trimers. High-speed AFM has even shown the dynamics of the protein lattice [40]. Protein immobility has also been measured for annexin bound plasma membrane nanotubes indicating that protein lattices can also form in highly curved regions. The functional role of these lattices in membrane repair remains unknown, however it was recently shown that ANXA5 can change the physical properties of the membrane [39]. Additionally, another function could be to provide friction to the membrane and stabilize the membrane rupture against further expansion [41].

## 6. Approaches to Inflict Damage to the Plasma Membrane

**Figure 4:**
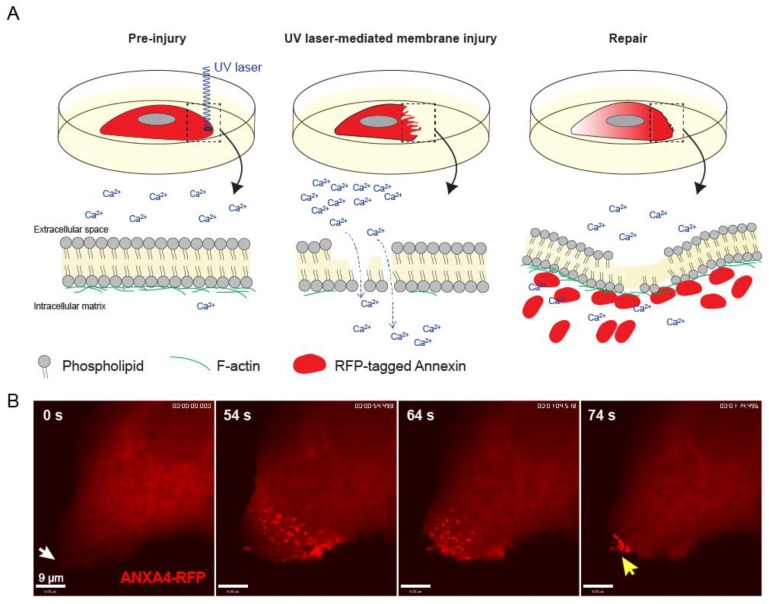
Plasma membrane injury inflicted by UV-laser ablation injury. (**A**) Schematic of ablation laser injury to monitor annexin behavior during plasma membrane repair. Ca^2+^ influx through the wounded membranes activates and enables annexin to bind and seal the hole by bending membrane and glue membrane edges together. (**B**) Sequential images from time-lapse movie of a MCF-7 breast carcinoma cell showing translocation behavior of ANXA4-RFP to the site of damage (white arrow) upon laser injury.

Many cell studies aimed at studying plasma membrane resealing use ablation laser to inflict spatial damage to the cell membrane. Here, a single and localized wound can be induced at the plasma membrane of cultured cells and repair can be followed using fast time-lapse imaging [13,41,42]. The main advantages include user’s control over the extent of damage by adjusting the laser power and the ability to assess cellular repair kinetic and monitor the action of fluorescently-tagged proteins involved in repair (Figure 4) [43]. A possible drawback is that membrane damage triggered by UV-ablation laser induces local high temperatures at the injured membrane, which can potentially affect membrane proteins and lipids and create thermally-induced diffusion and denaturation artifacts. However, we and others have demonstrated tempo-spatial recruitment of repair proteins occurs within 10–45s of laser-induced plasma membrane injury [28,44], indicating that the repair machinery is not disabled by the collateral thermal damage. Importantly, complimentary methods to induce membrane injury, such as the use of glass bead injury, detergents, and scraping, show that the same repair proteins are recruited to damaged membrane (including annexins, actin, ESCRT III) as with laser injury and needed for repair [14,45]. This suggests that laser-induced plasma membrane injury can provide insight into repair mechanisms that are of biological significance, since all injuries (artificial and, potentially, physiological/pathological) converge the point of Ca^2+^ influx, which is the key stimulus for plasma membrane repair mechanisms.

An alternative method for inflicting cell injury is to use thermoplasmonic [46]. Irradiation of plasmonic nanostructures using near infrared (NIR) light results in an extremely localized temperature increase which can be used to disrupt plasma membranes. This strategy has been used for fusion of cells [47] and membrane vesicles [48]. Both pulsed lasers or continuous wave lasers can be used in combination with plasmonic nanoparticles for disrupting membranes [49,50,51,52]. We envision that this technique will provide a fruitful approach for investigation of plasma membrane repair in the future in particular when combined with predictions from theoretical calculation on the stability of membrane holes decorated with annexins scaffolds.

## 7. Concluding Remarks

This synergy fostered by interdisciplinary research achieves its full potential when different perspectives are integrated to comprehensively understand a complex cellular phenomenon. Here, we describe the development of new complementary strategies that, when combined, have elucidated mechanisms underlying membrane shaping and repair. Our recent findings, which could only be revealed through interdisciplinary collaboration, show that there is more to annexins than previously anticipated. In particular there are many unanswered questions in relation to the complex interaction between annexins and curved membranes, and how physical cues aid in membrane healing. We surmise that, a combination of novel membrane assays and high resolution imaging together with multiscale simulations will provide major progress in resolving the enigmatic process of membrane repair in the future.
